# On Reverse Shrinkage Effects and Shrinkage Overshoot

**DOI:** 10.1007/s11336-022-09872-8

**Published:** 2022-06-05

**Authors:** Pascal Jordan

**Affiliations:** University of Hamburg, Von-Melle-Park 5, 20146 Hamburg, Germany

**Keywords:** shrinkage, prior, MAP, Lagrange multiplier, educational testing

## Abstract

Given a squared Euclidean norm penalty, we examine some less well-known properties of shrinkage estimates. In particular, we highlight that it is possible for some components of the shrinkage estimator to be placed further away from the prior mean than the original estimate. An analysis of this effect is provided within three different modeling settings—encompassing linear, logistic, and ordinal regression models. Additional simulations show that the outlined effect is not a mathematical artefact, but likely to occur in practice. As a byproduct, they also highlight the possibilities of sign reversals (“overshoots”) for shrinkage estimates. We point out practical consequences and challenges, which might arise from the observed effects with special emphasis on psychometrics.

**Supplementary Information:**

The online version contains supplementary material available at 10.1007/s11336-022-09872-8.

## Introduction

The effect of shrinkage or of “pooling of prior and likelihood information” is an essential part of hierarchical models (Gelman & Hill, Gelman and Hill (2007)). A traditional way to introduce the concept of shrinkage in its simplest form is the prediction of an unknown random variable based on data and prior knowledge (see chapter 7 of Searle, Casella, & McCulloch, 2006). For instance, given IQ test scores (data y) of John, we might be interested in predicting John’s true general IQ based on an underlying model and prior information. The model could consist of assuming normally distributed test scores around the true score *f* and the prior would ideally match with the distribution of the true scores in the population. Given this setup, the likelihood-based inference $\hat{f}$ (MLE) could potentially be improved in terms of expected squared error loss by incorporating the prior information, see also the closely related topic of ridge regression (Hoerl & Kennard, 1970a), or the general topic of best prediction, as outlined in Searle et al. (2006). Moreover, the resulting estimate turns out to be a compromise between (respectively a weighted average of) the likelihood-based estimate and the best a priori prediction (see p.233 in Lehmann & Casella, 1998). This in turn justifies the use of the term “shrinkage” when discussing the effect of applying this procedure. In addition, it is in line with our intuitive expectation. That is, by incorporating the prior information we expect the estimate to tend toward the prior mean.

**Fig. 1:**
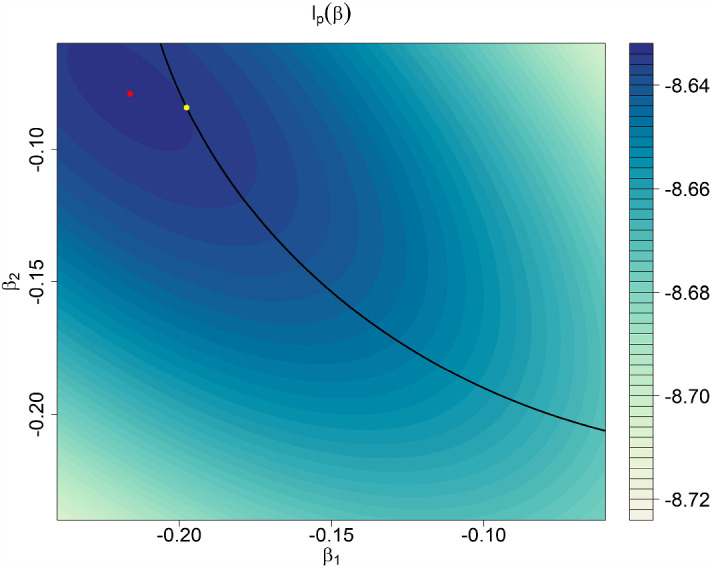
Illustration of the “reverse shrinkage” effect in a contour plot corresponding to a normal linear model likelihood. Shown are the MLE (in red) as well as the Bayesian MAP (in yellow) using independent priors with a common precision parameter. The second component (*y*-coordinate) of the MAP is larger in magnitude than the respective MLE component (amplification effect) (Color figure online).

Now assume the following slightly altered framework: John solves the items of an IQ test which is now supposed to measure two dimensions (say: numerical and verbal IQ). Based on his performance he scores 112 and 104 on the numerical and verbal component, respectively. As in the former unidimensional setup, we introduce a prior on the dimensions, which, for the sake of simplicity, is independent normal and centered around 100. By the same token as in the unidimensional case, we expect shrinkage of the likelihood-based estimates toward their prior mean. However, the resulting estimates turn out to be 108 and 106, so that John’s estimated verbal IQ is further away from the prior mean than the purely likelihood-based estimate. In other words, rather than shrinkage, we observe amplification of the distance from the prior mean (“reverse shrinkage”—see Sect. 2, case 1 for a theoretical and Sect. 4 Fig. 1 for a graphical explanation). In fact, we may stretch this example as follows: Suppose Bob scored 105 and 99 in the numerical and verbal IQ domain, respectively. After introducing the prior knowledge, i.e., abilities centered around 100, we may end up with estimates of 103 and 101—placing Bob above average on the verbal IQ domain despite the fact that the MLE indicated performance below average.

**Fig. 2:**
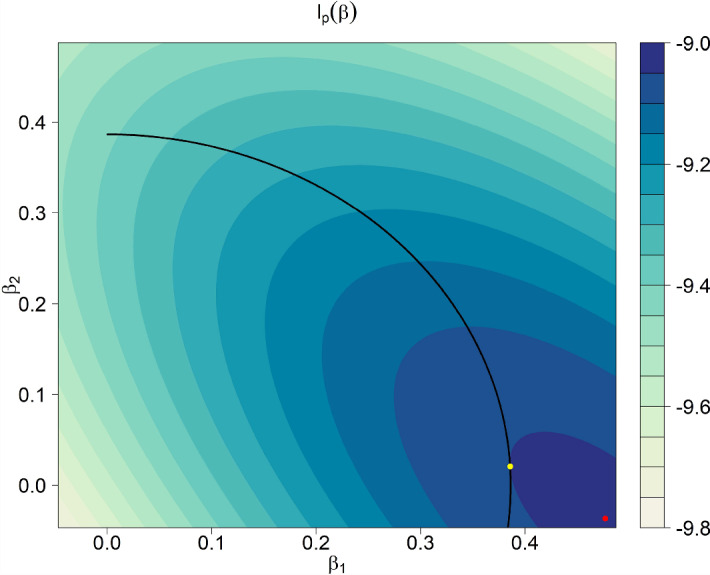
Illustration of the “shrinkage overshoot” effect in a contour plot corresponding to a normal linear model likelihood. Shown are the MLE (in red) as well as the Bayesian MAP (in yellow) using independent priors with a common precision parameter. The signs of the second components (*y*-coordinates) of the MAP and the MLE differ (sign reversal effect) (Color figure online).

The aim of this paper is to highlight that the described reverse shrinkage pattern is not based on an artificially constructed counterexample, but is likely to arise when using prior information in a multidimensional setting. To this end, we will provide analytical results of the reverse shrinkage effect in different modeling classes as well as some simulation results. Whereas the analytical results (Sect. 2) point out the possibility of such a previously introduced “amplification” effect (i.e., the existence), they leave open the question as to whether the effect is likely to occur in practical applications of shrinkage based models. To address the latter, some simulations (Sect. 3) are included which highlight that the described “amplification” effect is not a mathematical artefact. In conjunction with this, we will also point out the possibility of “sign reversals” (“shrinkage overshoot”—see Sect. 3 for examples and Fig. 2 in Sect. 4 for a graphical explanation), i.e., the possibility that the likelihood-based estimate may be below the prior mean, yet, the corresponding “shrinkage” estimate lies above the prior mean—like in the example of Bob. Finally, a graphical explanation of these counterintuitive effects will be given (Sect. 4) and practical problems which arise from the effect(s) will be outlined in Sect. 5.

## Theoretical Analysis

In this section, we point out various conditions under which the previously described phenomenon of amplification can be established. We first examine a model which underlies the IQ test scoring example—namely a linear model with offset (Case 1). Subsequently, we analyze the effect in the context of ridge regression (Case 2). We then aim at broadening the scope by analyzing the effect within a more general class of models—including logistic regression (Case 3). In all cases, we focus on a formulation of the shrinkage effect via a sum of squares penalty function, i.e., if $\beta$ denotes the model parameter vector, the penalty term will always be of the type $t \sum _i \beta _i^2$ for some fixed shrinkage parameter *t*. However, we also include a discussion of partially flat priors—that is, we examine the effect of a partial penalty of the type $t \sum _{i \in I} \beta _i^2$, wherein the summation does not extend over all coefficients (which in a Bayesian setup is interpretable in terms of using a flat prior for those components not appearing in the summation). Finally, although our paper primarily deals with the analysis of the impact of shrinkage on the latent ability estimate of an individual test taker, we also highlight connections to the predictions of group-level effects in a linear mixed model framework.

### Case 1: Linear Model with Offset

Historically (e.g., Spearman, 1904; Thomson, 1951; Steiger, 1979) and contemporarily (see, e.g., Canivez & Watkins, 2010), IQ tests have been closely linked to the linear factor analysis model. The latter model resembles a linear regression model (see ch. 9 of Mardia, Kent & Bibby, 1979) when it comes to the estimation of the person parameters (factors) assuming item parameters (e.g., loadings) are known^1^ from a previous test calibration with a sufficiently large sample size. Hence, a linear model with offset is the natural starting point to analyze the IQ-test framework. More specifically, given known item difficulties $\mu _i$ (for $i=1,\dots , k$) and a known $k \times p$ full column rank matrix $\Lambda$ of factor loadings, the linear model type decomposition

$$\begin{aligned} y=\mu +\Lambda f + \epsilon \end{aligned}$$

is assumed, wherein the $p \times 1$ vector of factors *f* entails the unknown person parameters which are to be estimated from observing the data *y*. The *i*-th component of *y* denotes the score of the test taker in a particular subtest, for instance, his/her score in a number division task which is part of the overall numerical IQ scale. The usual modeling assumptions presume normally distributed error terms with zero means and zero covariances (conditional on *f*).

Although this represents a commonly used notation in the psychometric framework, we prefer to use the standard regression notation in order to unify the notation with the one that will be used in later sections. Therefore, we will in the following use the regression design matrix *X* in place of $\Lambda$ and the vector of unknown regression coefficients $\beta$ in place of *f*. Without loss of generality, we will further also assume offsets $\mu _i=0$ in order to simplify the formulas. Hence, we write

$$\begin{aligned} y=X\beta +\epsilon , \qquad \epsilon |\beta \sim N (0,diag(\sigma ^2_i)_{i=1,\dots , k}), \end{aligned}$$

wherein in the IQ testing example $\beta$ contains two components, one numerical and one verbal IQ component. For the subsequent derivations we also fix the error variances to a common value $\sigma ^2_i=\sigma ^2$. The remarks following the derivations will, however, clarify that the results also hold for the general case of unequal measurement error variances.

The maximum a posteriori estimate (MAP) using independent normal priors with common precision, i.e., assuming $\beta \sim N(0,\frac{\sigma ^2}{t}I)$, is given by the expression:

1 $$\begin{aligned} \hat{\beta }(t):=(X^TX+tI)^{-1}X^Ty. \end{aligned}$$

Note that the MLE is contained in this expression via setting $t=0$. More generally, the posterior distribution of the regression parameter $\beta$ is given by (see Hsiang, 1975)

$$\begin{aligned} \beta |y \sim N\left( (X^TX+tI)^{-1}X^Ty, \sigma ^2 \left( X^TX + t I\right) ^{-1}\right) \end{aligned}$$

showing that all of the traditionally used Bayesian estimators (e.g., the expected a-posteriori estimator) coincide with the MAP.

As we will be concerned with analyzing the behavior of the components of the MAP when we change the level of shrinkage via *t*, we need the derivative of $\hat{\beta }(t)$, which (with $A(t):=X^TX+tI$) is given by:

2 $$\begin{aligned} \hat{\beta }'(t)= & {} -A(t)^{-1}\frac{\partial A}{\partial t} (t)A(t)^{-1}X^Ty=-(X^TX+tI)^{-1}I(X^TX+tI)^{-1}X^Ty\nonumber \\= & {} -(X^TX+tI)^{-2}X^Ty. \end{aligned}$$

As in the example of John, assume now that the person scored such that the estimated factor scores, using a specific level of shrinkage *t* (in the example $t=0$), are above (or more precisely: not below) the prior mean, i.e., assume that each component of $\hat{\beta }(t)$ is nonnegative. Then, depending on the response pattern *y*, we will show that it is possible for John to “gain" additional points on some dimension—hence placing him even further away from the population mean—by using a stronger prior precision, that is, a higher level of shrinkage.

Let $e_i$ denote the *i*-th unit vector. If we can prove that there is a response *y* such that $e_j^T(X^TX+tI)^{-1}X^Ty \ge 0$ holds for $j=1, \dots , p$ and with the additional property $g_0(y)=e_i^T(X^TX+tI)^{-2}X^Ty<0$ for some component *i*, then the first property will ensure that the MAP estimates are above (not below) the prior mean, while the second property will ensure via (2) that the *i*-th component increases with increasing level of shrinkage.

#### Theorem 1

Let *X* denote a $(k \times p)$ matrix of full column rank. Let $t\ge 0$ denote a penalty parameter. If the *i*-th column of the matrix $(X^TX+tI)^{-1}$ contains a negative entry, then there is a response vector $y^*$ such that the following holds

- $\hat{\beta }(t):=(X^TX+tI)^{-1}X^Ty^*$ consists of nonnegative entries.
- There exists a $t'>t$ such that $\hat{\beta _{i}}(t')>\hat{\beta }_{i}(t)\ge 0$.

#### Remark

In the educational testing framework, (*a*) and (*b*) imply that there is a response vector such that the test taker’s inferred abilities are not below average on each dimension, and, such that increasing the shrinkage toward $t'$ further increases the distance between the inferred ability on the *i*-th dimension and the population mean.

#### Proof

It needs to be shown that there is a response vector *y* such that (1) is (componentwise) nonnegative while the *i*-th component of (2) is positive. To this end, we examine conditions under which for every response vector with

$$\begin{aligned} e_j^T (X^TX+tI)^{-1}X^Ty\ge 0 \quad \forall j \end{aligned}$$

we also have

$$\begin{aligned} e_i^T (X^TX+tI)^{-2}X^Ty\ge 0. \end{aligned}$$

In more technical terms, we examine conditions under which the inequality

3 $$\begin{aligned} e_i^T (X^TX+tI)^{-2}X^Ty\ge 0 \end{aligned}$$

is a consequence^2^ of the system of inequalities

4 $$\begin{aligned} e_j^T (X^TX+tI)^{-1}X^Ty\ge 0 \quad \forall j. \end{aligned}$$

According to Farkas’ Lemma (see appendix), (3) is a consequence of the system of inequalities (4) if and only if the vector $e_i^T (X^TX+tI)^{-2}X^T$ can be expressed as a nonnegative linear combination of the set of vectors $(e_j^T (X^TX+tI)^{-1}X^T)_{j=1, \dots p}$ The latter condition means that there is a nonnegative vector $\lambda$ such that

$$\begin{aligned} X(X^TX+tI)^{-2}e_i = X(X^TX+tI)^{-1}\lambda \end{aligned}$$

holds, or, equivalently:

$$\begin{aligned} X(X^TX+tI)^{-1}\left( (X^TX+tI)^{-1}e_i - \lambda \right) =0. \end{aligned}$$

Due to the assumption of a design matrix *X* of full column rank the above equation may be rewritten as

$$\begin{aligned} (X^TX+tI)^{-1}e_i = \lambda \end{aligned}$$

To conclude, we have arrived at the following: If the *i*-th column of the matrix $(X^TX+tI)^{-1}$ contains at least one negative entry, then the inequality (3) is *not* a consequence of the system of inequalities (4). The latter then implies that there has to exist a response vector $y^*$such that the system of inequalities (4) holds, but the inequality (3) is violated. This response vector can then be characterized as a response vector ensuring nonnegative estimates (i.e., each component is not below the prior mean of zero) while exhibiting a higher estimate for the *i*-th component when increasing the shrinkage level. $\square$

As *i* in the statement of Theorem (1) was arbitrary, we can conclude the following: If the matrix $(X^TX+tI)^{-1}$ has at least one negative entry, then the corresponding column index provides a component for which there exists a response vector $y^*$ which is scored above the prior mean and with the claimed amplification property on some dimension. Hence, it all boils down to examine when the matrix $(X^TX+tI)^{-1}$ consists of only nonnegative entries. We will examine this in the two-dimensional setting of the example and then provide some results for the general case. Before, however, further discussing these issues, we note some important generalizations of the above analysis.

Generalizations:

- *Unequal error variances* The same analysis applies in case of unequal variance for the error terms. The formulas still hold if the terms $X^TWX$ and $X^TWy$ are substituted for $X^TX$ and $X^Ty$, respectively. To explicitly state the result: For the existence of a response vector with the amplification property, it is necessary and sufficient that the matrix $(X^TWX+tI)^{-1}$ contains at least one negative entry.
- *Prior correlations* We can further generalize the result by introducing prior dependencies between the latent factors. That is, if we substitute a known prior covariance matrix $\Sigma$ (up to a common precision factor *t*) for the identity matrix *I*, we get the following result: In order that there exists a response vector with the amplification property (on some dimension), it is necessary and sufficient that the matrix $\Sigma ^{-1}(X^TWX +t\Sigma ^{-1})^{-1}$ contains at least one negative entry.
  Note that if we use this prior specification, i.e., $\beta \sim N(0, \frac{\sigma ^2}{t}\Sigma )$, then the posterior (see also Hsiang, 1975) becomes:
  $$\begin{aligned} \beta |y \sim N\left( (X^TWX+t\Sigma ^{-1})^{-1}X^TWy, \sigma ^2 \left( X^TWX + t \Sigma ^{-1}\right) ^{-1}\right) \end{aligned}$$
- *Bayesian estimates* The computed estimate is identical with a Bayesian maximum a-posterior (MAP) estimate. However, due to the symmetry of the involved distributional (here: normality) assumptions, the posterior is symmetric. Hence, the expected a-posterior estimate is identical to the MAP. Therefore, the analyzed effect is also deducible in the integration (EAP) domain and not confined to the maximization-based setup. Furthermore, using the rationale that the skewness of a posterior distribution tends to decrease with the amount of sample data, we may arrive at the conjecture that in a large sample size setting, the EAP will always behave qualitatively as the MAP (which will be relevant when we change the setup of analysis in Case 3).
- *Relaxing the nonnegativity restriction* Note that we might have an amplification effect even if $(X^TX+tI)^{-1}$ consists of nonnegative entries. Although this seems to contradict the theorem, note that we required nonnegative estimates on each component in the theorem. Therefore, if $(X^TX+tI)^{-1}$ is nonnegative, then we may only infer that there is no response leading to nonnegative estimates on each component and showing the amplification property. However, if we relax this condition, then we may generalize the effect in an important way. The statement and proof of this generalization is given below.

#### Theorem 2

Let *X* denote a $(k \times p)$ matrix of full column rank. Let $t\ge 0$ denote a penalty parameter. If the *i*-th column of the matrix $(X^TX+tI)^{-1}$ is not a (positive) multiple of the *i*-th unit vector $e_i$, then there is a response vector $y^*$ such that the following holds:

- $$\hat{\beta }_i(t):=e_i^T(X^TX+tI)^{-1}X^Ty^*\ge 0$$.
- There exists a $t'>t$ such that $\hat{\beta _{i}}(t')>\hat{\beta }_{i}(t)\ge 0$.

#### Proof

We may again resort to Farkas’ Lemma. The inequality $e_i^T (X^TX+tI)^{-2}X^Ty\ge 0$ is a consequence of the “system” of inequalities $e_i^T (X^TX+tI)^{-1}X^Ty\ge 0$ if and only if there is a nonnegative scalar $\lambda$ such that

$$\begin{aligned} X(X^TX+tI)^{-2}e_i = X(X^TX+tI)^{-1}e_i \lambda . \end{aligned}$$

Using the same arguments as in the proof of Theorem 1 this may be reduced to having

$$\begin{aligned} (X^TX+tI)^{-1}e_i = \lambda e_i \end{aligned}$$

which means that the *i*-th column is a multiple of the *i*-th unit vector. $\square$

#### Remark

Again, this result allows for a generalization (with straightforward changes in the proof) by introducing prior dependencies between the latent factors and by also considering unequal measurement error variances. If the *i*-th column of the matrix $\Sigma ^{-1}(X^TWX + t\Sigma ^{-1})^{-1}$ is not a positive multiple of the *i*-th unit vector, then it is possible to deduce amplification.

#### The Two-Dimensional Educational Testing Example

Assume the test is two-dimensional and that each ability contributes positively to the solving of the items, i.e., assume a matrix of positive factor loadings. Then, the cross product matrix $X^TX$ contains only positive entries *a*, *b*, *d* and we have

$$\begin{aligned} X^TX+tI=\begin{pmatrix} a+t &{}\quad b \\ b&{} \quad d+t \end{pmatrix} \end{aligned}$$

with the inverse given by

$$\begin{aligned} (X^TX+tI)^{-1}=\frac{1}{\text {det}(X^TX+tI)}\begin{pmatrix} d+t &{} \quad -b \\ -b &{} \quad a+t \end{pmatrix}. \end{aligned}$$

Clearly, each column contains a negative entry and we may therefore conclude that for each (intelligence) factor there is always a response vector leading to estimates not below average on each dimension and such that increasing the shrinkage parameter increases the distance from the prior mean on the specific factor. In the simulation (see Sect. 3), it is shown that the set of responses *y* with such a property is not a negligible small set.

If we allow for prior correlations, the analysis becomes more complicated. According to the remarks on generalizations following Theorem 1, we need to examine the matrix $\Sigma ^{-1}(X^TX + t\Sigma ^{-1})$ in place of the former expression $(X^TX+tI)^{-1}$. Using the equality

$$\begin{aligned} \Sigma ^{-1}(X^TX + t\Sigma ^{-1})^{-1}= (X^TX\Sigma + tI)^{-1} \end{aligned}$$

and the abbreviations $\sigma _{i,j}$ for the (*i*, *j*)-th entry of the matrix $\Sigma$, we can write:

$$\begin{aligned} (X^TX\Sigma + tI)^{-1} = \begin{pmatrix} b\sigma _{1,2}+d\sigma _{2,2}+ t &{} \quad -( a\sigma _{1,2}+b\sigma _{2,2}) \\ -(b\sigma _{1,1} + d\sigma _{12}) &{} \quad a\sigma _{1,1}+b\sigma _{1,2}+ t \end{pmatrix} \end{aligned}$$

As an illustrative example, we assume simple structure, i.e., an orthogonal design matrix such that $b=0$ holds. It can then be seen that any positive prior correlation leads to a negative entry in each column. Hence, under positive prior correlation, we can always find a response vector that is scored not below average on each dimension and such that increasing the shrinkage parameter increases the estimate of the first (or second) dimension. Further, if we restrict ourselves to the effect underlying Theorem 2, then we may deduce the following: Any nonzero prior correlation (we still assume simple structure) leads to a shrinkage effect as described in Theorem 2. That is, we can find a response vector that is scored not below average on the *i*-th dimension and such that increasing the shrinkage level increases the estimate on the *i*-th dimension further.

The examination of the general case, i.e., not requiring simple structure, is more complicated for the effect described in Theorem 1. However, the effect depicted in Theorem 2 is still straightforward to detect: Unless the first (second) row of the crossproduct matrix is orthogonal to the second (first) row of the prior correlation matrix, the shrinkage effect of the type stated in Theorem 2 can be deduced for the second (first) dimension.

#### A General result for Nonnegative Factor Matrices

It is impossible to construct a response vector with paradoxical shrinkage behavior (as stated in Theorem 1) if and only if the inverse of the matrix $(X^TX+tI)^{-1}$ contains only nonnegative entries. Given nonnegative factor loadings, the latter will, however, only be the case if and only if (see, e.g., Lemma 12 in Jordan and Spiess, 2012) the matrix of factor loadings is of simple structure, i.e., contains no cross-loadings.

##### Corollary 1

If the columns of *X* are orthogonal and if the normal prior specifies independence between the components of $\beta$ (as assumed in Theorem 1), then the model is not prone to reverse shrinkage effects.

Hence, within every factor analysis model with nonnegative factor loadings and not of simple structure, the existence of a response vector which gives rise to the amplification phenomenon is guaranteed. Again, the simulation results in Sect. 3 provide the additional information that these types of response vectors do not form a thin set, but may emerge with substantial probability.

With respect to Corollary 1, we emphasize the necessity of an independence prior. That is, if we introduce correlations in the prior, then reverse shrinkage effects can occur even in a simple structure model—see the analysis depicted in the previous subsection.

#### Response Vectors Leading to Amplification

Until now, we discussed conditions under which the amplification property occurs. Yet, the theorems did not provide direct clues on how to find a response vector $y*$ which leads to the amplification phenomenon. Here we provide an informal interpretation of the properties of such a response vector. We restrict our discussion to an amplification on the first dimension with the simultaneous requirement that all estimates are not below the prior mean. Other cases can be deduced accordingly. To this end, we look at the requirement of amplification on the first dimension, namely the inequality

$$\begin{aligned} e_1^T(X^TX+tI)^{-2}X^Ty<0 \end{aligned}$$

which guarantees amplification of the first component—provided we also have a nonnegative estimate in the *i*-the component. We may rewrite the above inequality as

$$\begin{aligned} e_1^T(X^TX+tI)^{-1}\hat{\beta }(y)<0, \, \, \hat{\beta }(y)=(X^TX+tI)^{-1}X^Ty \end{aligned}$$

For the special case of the MLE, which we will examine first, we have

$$\begin{aligned} e_1^T(X^TX)^{-1}\hat{\beta }(y)<0, \, \, \hat{\beta }(y)=(X^TX)^{-1}X^Ty \end{aligned}$$

According to standard results in the linear model, the matrix $(X^TX)^{-1}$ contains the estimated variances and covariances of the regression parameter estimates, that is, the entry in the *j*-th row and *l*-th column is equal to the covariance of $\hat{\beta }_j$ and $\hat{\beta }_l$. Further, suppose we are interested in the estimated covariance between $c_1^T \hat{\beta }$ and $c_2^T \hat{\beta }$. Then this covariance may be computed as follows:

5 $$\begin{aligned} c_1^T(X^TX)^{-1}c_2. \end{aligned}$$

With this in mind, let now *D* denote the set of vectors $c_2$ which give rise to linear combinations with a negative covariance with $\hat{\beta }_1$, i.e., let $D:=\{c_2 \in {\mathbb {R}}^p|\, e_1^T(X^TX)^{-1}c_2<0\}$. Geometrically, this set of vectors may be described by the set of vectors which lie in the corresponding open half-space determined by a hyperplane (through the origin) with normal vector $e_1^T(X^TX)^{-1}$. As such, this set is a convex cone, i.e., it is closed under addition and positive scalar multiplication.

With this terminology, we may now characterize the set of response vectors which give rise to the amplification property as follows: A response vector which gives rise to nonnegative estimates ($\hat{\beta }(y)\ge 0$) shows amplification on the first component if and only if the estimate, when viewed as weights for a linear combination, corresponds to a vector of *D*. If such an element of *D* were used a-priori, then it would correspond to a linear combination of the regression parameters with negative covariance with the estimate of the first component. Note that we used the term “a-priori,” because it is not true that the covariance of $\hat{\beta }_1$ with the linear combination given by setting $c_2:=\hat{\beta }$ is computable via formula (5). The reason is that in (5) $c_2$ (and $c_1$) is supposed to be a vector of weights which are independent of the data *y*, whereas by choosing a vector of weights of the form $c_2:=\hat{\beta }(y)$ we introduce dependency on the data. Therefore our rather contrived formulation above.

Overall, this provides an informal argument characterizing response vectors which introduce local amplification around the MLE.

We may apply the same reasoning around a nonzero initial value of *t*, i.e., for the MAP. However, the interpretation of $(X^TX+tI)^{-1}$ has to change. This matrix does not provide the estimated covariance matrix of the regression parameters anymore. However, it corresponds to the covariance matrix of the posterior distribution of the regression parameters in the corresponding Bayesian model. Except for this change in interpretation, the same reasoning as above can now be applied to this Bayesian setting.

### Case 2: Linear Model with Centered Predictors

If instead of a linear model with offset, a linear model with intercept $\beta _0$ and centered predictors is given, then, assuming priors $\beta \sim N(0,\frac{\sigma ^2}{t}I), f(\sigma ^2) \propto \frac{1}{\sigma ^2}$ and an (improper) flat prior for the intercept, i.e., $\beta _0 \sim U(-\infty ,\infty )$, the MAP of the regression coefficients for the predictors may be written as

$$\begin{aligned} \hat{\beta }(t)=(X^TX+tI)^{-1}X^Ty^c, \end{aligned}$$

wherein $y^c$ refers to the centering of the dependent variable. In order to adapt the approach of case 1, we have to account for the fact that the response vector *y* is now centered, i.e., has to satisfy $y^T1=0$ (with “1” denoting a vector of ones).

#### Theorem 3

Let *X* denote a $(k \times p)$ matrix of full column rank and with centered columns, i.e., satisfying $X^T 1 = 0$. Let $t\ge 0$ denote a penalty parameter. If the *i*-th column of the matrix $(X^TX+tI)^{-1}$ is not a (positive) multiple of the *i*-th unit vector $e_i$, then there is a response vector $y^*$ such that the following holds:

- $$\hat{\beta }_i(t):=e_i^T(X^TX+tI)^{-1}X^Ty^*\ge 0$$.
- There exists a $t'>t$ such that $\hat{\beta _{i}}(t') >\hat{\beta }_{i}(t)\ge 0$.
- $$y^*$$ is centered: $${y^{*}}^{T} 1 = 0$$.

#### Proof

We will examine conditions under which every *centered* response vector $y^c$ with corresponding *i*-th component of $\hat{\beta }$ not below prior mean, i.e., with $e_i^T(X^TX+tI)^{-1}X^Ty^c\ge 0$, also satisfies $e_i^T (X^TX+tI)^{-2}X^Ty^c\ge 0$ (derivative of the *i*-th component with respect to *t* is nonpositive). Stated in terms of the terminology underlying Farkas’ Lemma, we examine the following: Under which conditions is the inequality $e_i^T (X^TX+tI)^{-2}X^Ty\ge 0$ a consequence of the system of inequalities

$$\begin{aligned} e_i^T(X^TX+tI)^{-1}X^Ty\ge 0, \, \, 1^T y \ge 0, \, \, - 1^T y \ge 0 \, ? \end{aligned}$$

According to Farkas’ Lemma, the existence of nonnegative scalars $\lambda , \delta _1, \delta _2$ such that

$$\begin{aligned} e_i^T (X^TX+tI)^{-2}X^T = \lambda e_i^T(X^TX+tI)^{-1}X^T +\delta _1 1 + \delta _2 (-1) \end{aligned}$$

is a necessary and sufficient condition for the above implication. Transposing both sides and defining $\delta := \delta _1 - \delta _2$, the above equation reduces to:

$$\begin{aligned} X(X^TX+tI)^{-2}e_i = \lambda X(X^TX+tI)^{-1}e_i + \delta 1. \end{aligned}$$

Premultiplying with $X^T$, and using the fact that the predictors are centered, we deduce:

$$\begin{aligned} X^TX(X^TX+tI)^{-2}e_i = \lambda X^TX(X^TX+tI)^{-1}e_i, \end{aligned}$$

from which the claim follows analogous to the proof of Theorem 2. $\square$

Note that the above setup includes the classical ridge regression as a special case (using scaled predictor variables, in which case $X^TX$ reduces to the correlation matrix; see Hoerl & Kennard, 1970a; b). Moreover, in the simulation presented in Sect. 3 we use a classical ridge regression example to highlight the prevalence of the amplification property.

### Case 3: Log-Concave Likelihood Model with Positive Predictors

We now move away from the linear model and examine a type of model that includes various prominent regression models for categorical variables (e.g., logistic regression and cumulative logit-type ordinal regression). We will only specify the type of log-likelihood we are dealing with. To this end, assume that the log-likelihood^3^ may be written as

6 $$\begin{aligned} l(\beta ):=\sum _{i=1}^k l_i(x^T_i \beta ) \end{aligned}$$

for some twice continuously differentiable functions $l_i: {\mathbb {R}} \mapsto {\mathbb {R}}$ satisfying $l_i''<0$ throughout ${\mathbb {R}}$. For the sake of concreteness, we note that, for a linear model with known variances and offsets, we have $l_i(z)=-\frac{1}{2\sigma ^2_i}(y_i-\mu _i-z)^2$, and for a logistic regression model we have $l_i(z):=\mu _i+z-\log (1+e^{\mu _i +z})$ or $l_i(z):=-\log (1+e^{\mu _i +z})$ depending on whether the response $y_i$ was correct or incorrect. If we assume in addition a design matrix (with *i*-th row given by $x_i$) of full rank, then the above log-likelihood function is strictly concave—implying the uniqueness of the MLE (and also the uniqueness of the following MAP-extension).

We now add some normal prior knowledge controlled by a shrinkage/scaling parameter *t*, i.e., we assume that the log prior is (up to an additive constant independent of the parameter) given by

$$\begin{aligned} \gamma ^p(\beta ):=-\frac{1}{2}(\beta -\beta _0)^Tt\Sigma _p^{-1} (\beta -\beta _0), \end{aligned}$$

wherein the nonsingular matrix $\Sigma _p$ is fixed, and, wherein *t* acts as the analogue of the shrinkage parameter in cases 1 and 2. The corresponding log-posterior therefore equals $l(\beta )+\gamma ^p(\beta )$. In order to derive the MAP, the derivative of $l(\beta )+\gamma ^p(\beta )$ needs to be computed. Using the notation $u(t,\beta )$ to denote this derivative and to indicate at the same time the dependency on the shrinkage parameter *t*, we get:

7 $$\begin{aligned} u(t,\beta )= \sum _{i=1}^kl'_i(x_i^T\beta ) x_i-t\Sigma _p^{-1}(\beta -\beta _0). \end{aligned}$$

Setting $u=0$ implicitly defines the MAP as the solution of this equation. Using the implicit function theorem (e.g., Dontchev & Rockafellar, 2009, theorem 1B.1), the rate of change of the solution $\hat{\beta }(t)$ may be computed as (*D* denoting the differential):

8 $$\begin{aligned} D\hat{{\varvec{\beta }}}(t)=-\left( \sum _i x_i x_i^T l''_i (x_i^T\hat{\beta })-t\Sigma _p^{-1}\right) ^{-1} \Sigma _p^{-1}(\hat{\beta }-\beta _0). \end{aligned}$$

If all predictors are positive and if $\Sigma _p=I$, then the inverse appearing in (8) must contain a negative entry, say in the first row and second column. It is then possible for the dot product of that row with the vector $(\hat{\beta }-\beta _0)$ to become negative, provided the second component of $(\hat{\beta }-\beta _0)$ is sufficiently large positive. In that case, it follows that the first component of the MAP is increasing with increasing level of shrinkage. Of course, Eq. (8) shows that we may also have the same effect in the presence of nonzero correlations and not necessarily positive predictors. Again, the simulation in Sect. 3 provides various illustrations of this effect. Note, however, that the above reasoning does not provide a strict proof, as $\hat{\beta }$ cannot vary freely, but may be restricted in a complicated way. For example, in a logistic regression type model, $\hat{\beta }$ may take on at most $2^k$ different values. Nevertheless, the reasoning clearly depicts a similarity to the linear model and therefore suggests that we may expect qualitatively the same shrinkage effects as already deduced for the linear model. Finally, it should also be noted that we may further enlarge the modeling class by removing the assumption of positive predictors and by arguing via (8)—using the assumption that the matrix contains nonzero off-diagonal elements.

### Flat Priors

The previous cases demonstrated a reverse shrinkage effect for different multiparameter models. In one-parameter models, i.e., within the unidimensional setup, it is, however, clear that the effect of introducing the (normal) prior is always a shrinkage effect (e.g., Lehmann & Casella, 1998, p.233) and that reverse shrinkage cannot occur. Hence, it might be speculated that using a normal prior for a single parameter—say $\beta _1$—and otherwise (improper) flat priors for the remaining parameters could potentially avoid the reverse shrinkage effect in the multidimensional setting. Surprisingly, quite the opposite is true, as will be shown using results from Hooker et al. (2009) and Jordan and Spiess (2018). On a purely technical level, the following derivation resembles the derivation given in Jordan and Spiess (2018). However, as the content underlying the derivation is different—i.e., in Jordan and Spiess (2018) the focus is on the impact of changes in the responses on the MLE, whereas herein we focus on the effect of inducing penalties while keeping responses fixed—we provide the full argument adapted to our case of studying properties of shrinkage estimates.

We note in advance that in the following we always implicitly assume the existence of the MLE—although conditions ensuring the existence can be obtained via standard results in convex/variational analysis (e.g., using theorems 1.9 and 3.26 in Rockafellar and Wets 2009). We assume^4^$p=2$ (two-dimensional setting) and the scenario of case 3 (strictly log-concave likelihood with positive predictor variables). Then, the corresponding log-posterior can be written up to an additive constant as

$$\begin{aligned} \sum _{i=1}^k l_i(x_i^T \beta )-\frac{1}{2}t\beta ^2_1, \end{aligned}$$

wherein the term $-\frac{1}{2}t\beta ^2_1$ equals the penalty which is obtained by using the prior $\beta _1 \sim N(0,\frac{1}{t})$ and an improper,^5^ flat prior for $\beta _2$, i.e., $\beta _2 \propto 1$. The gradient of the log-posterior has to vanish at the optimal solution. That is, if $\beta ^s=(\beta ^s_1,\beta ^s_2)$ denotes the Bayesian MAP, then we must have:

9 $$\begin{aligned} \sum _i l'_i(x_i^T \beta ^s)x_i -t(\beta ^s_1,0)^T=0. \end{aligned}$$

Likewise, the MLE, denoted as $\beta ^l=(\beta ^l_1,\beta ^l_2)$, has to satisfy

10 $$\begin{aligned} \sum _i l'_i(x_i^T \beta ^l)x_i=0. \end{aligned}$$

In the following we assume that both components of the MLE are above the prior mean of zero.

Subtracting (10) from (9) leads to the requirement

11 $$\begin{aligned} \sum _i \left( l'_i(x_i^T \beta ^s)-l'_i(x_i^T \beta ^l)\right) x_i -t(\beta ^s_1,0)^T=0. \end{aligned}$$

The key observation to note here is that if we had a shrinkage effect on both components, then the two vectors $\beta ^s$ and $\beta ^l$ would have to be ordered in the sense of the partial ordering in ${\mathbb {R}}^2$. However, from Eq. (11) we can rule out the possibility that $\beta ^s$ and $\beta ^l$ are ordered. For the latter we can argue by contradiction: Assume that $\beta ^s$ and $\beta ^l$ are ordered. More specifically, assume that $\beta ^s<\beta ^l$ holds, wherein the ordering refers to the partial ordering in ${\mathbb {R}}^2$ (the case $\beta ^s>\beta ^l$ can be treated similarly). As every predictor $x_i$ is positive, we then have $x_i^T \beta ^s<x_i^T \beta ^l$ for all *i* and as $l'_i$ is a strictly decreasing function (recall the assumption $l_i''<0$), we conclude that each term of the sum appearing in (11) is positive. The latter contradicts the fact that the second component of the left hand side of (11) must vanish. Hence, the initial assumption of ordered parameter estimates was false and we arrive at the result that this setting (positive predictors, flat priors) always entails a reverse shrinkage effect. Thus, for *every* response pattern (such that the MLE exists), we can conclude that some component of the MLE is closer to zero than the corresponding component of the shrinkage estimate.

### Shrinkage Overshoot

Up to now we have primarily examined the case of amplification on some component, i.e., we presupposed $\hat{\beta }_i\ge 0$ on all (or some) dimensions and examined under which conditions an increase in the shrinkage parameter places the estimate on a chosen dimension *i* further away from the prior mean of zero. However, we may also arrive at a second phenomenon which was introduced in the example of Bob in Sect. 1. In this case, we observed a performance below the prior mean and no amplification when applying shrinkage. Yet “improper” shrinkage was observed, as the estimate of Bob’s latent ability was placed above the prior mean after applying shrinkage.

We now turn to an analysis of this phenomenon:

#### Theorem 4

Let *X* denote a $(k \times p)$ matrix of full column rank. Let $t\ge 0$ denote a penalty parameter. If the *i*-th column of the matrix $(X^TX+tI)^{-1}$ is not a (positive) multiple of the *i*-th unit vector $e_i$, then for every penalty $t'>t$ there is a response vector $y^*$ such that the following holds

- $$\hat{\beta }_i(t'):=e_i^T(X^TX+t'I)^{-1}X^Ty^*<0$$.
- $$\hat{\beta }_i(t):=e_i^T(X^TX+tI)^{-1}X^Ty^*\ge 0$$.

#### Proof

We examine under which condition the inequality $e_i^T(X^TX+t'I)^{-1}X^Ty\ge 0$ is a consequence of the inequality $e_i^T(X^TX+tI)^{-1}X^Ty \ge 0$. According to Farkas’ Lemma, this holds if and only if there is a nonnegative scalar $\lambda$ such that

$$\begin{aligned} X(X^TX+t'I)^{-1}e_i = \lambda X (X^TX+tI)^{-1}e_i \end{aligned}$$

As *X* has full column-rank, this reduces to

$$\begin{aligned} (X^TX+t'I)^{-1}e_i = \lambda (X^TX+tI)^{-1}e_i. \end{aligned}$$

Using the notation $v:=(X^TX+tI)^{-1}e_i, v':= (X^TX+t'I)^{-1}e_i$, note that *v* may be characterized as the solution to’

12 $$\begin{aligned} (X^TX+tI)v = e_i. \end{aligned}$$

Now, due to the above reasoning, in order that the inequality $e_i^T(X^TX+t'I)^{-1}X^Ty\ge 0$ is a consequence of the inequality $e_i^T(X^TX+tI)^{-1}X^Ty \ge 0$, $v'$ must be a nonnegative multiple of *v* ($v':=\lambda v$). Further, $v'$ is characterized as the solution of the equation:

$$\begin{aligned} (X^TX+t'I)v' = e_i. \end{aligned}$$

Expanding the left side and using $v'=\lambda v$ and property (12), we arrive at:

13 $$\begin{aligned} (X^TX+t'I)v'= (X^TX+tI+(t'-t)I) \lambda v = \lambda (e_i + (t'-t)v)=e_i \end{aligned}$$

Hence, *v* needs to be a multiple of $$e_i$$ in order for the above equation to hold. Using the definition of *v*, i.e., $$v:=(X^TX+tI)^{-1}e_i$$, we therefore arrive at the condition that the *i*-th column of $$(X^TX+tI)^{-1} $$ must be zero except for its *i*-th entry. $$\square $$

### Outlook: Predicting Random Effects in a Linear Mixed Model

Interestingly, we can transfer some results on the amplification effect to a discussion of the prediction of random effects in a Linear Mixed Model (LMM). To this end, we follow the notation in Searle, Casella and McCulloch (2006) and write the basic equation of the LMM according to:

14 $$\begin{aligned} y=X\beta + Zu + \epsilon \end{aligned}$$

with *Z* denoting a fixed design matrix for the random effects and with *u* denoting the vector of all random effects. It is assumed that the vector of errors $\epsilon$ is independent of the random effects and that its covariance matrix is given by $\Sigma _{\epsilon }= \sigma ^2 I$. Further, for the vector of random effects *u* we denote its covariance matrix as $\Sigma _u$. All covariance matrices are assumed to be positive definite.

Given this setup, the best linear prediction of the random vector *u* based on data *y* can be computed by the following formula^6^ (see ch. 7 of Searle, Casella and McCulloch, 2006):

15 $$\begin{aligned} BLP(u)(y)= \mu _u + \Sigma _{u,y}\Sigma _{y,y}^{-1}(y-\mu _y) \end{aligned}$$

We have $\mu _u=0$ and we may further, without loss of generality, assume $\mu _y=0$ (i.e., $\beta =0$) in the following. According to the model equation (14), we may compute the two key quantities appearing in (15) as follows:

$$\begin{aligned} \Sigma _{u,y} = \Sigma _uZ^T, \, \, \Sigma _{y,y} = Z \Sigma _uZ^T + \sigma ^2 I. \end{aligned}$$

We now parametrize $\Sigma _u = \frac{1}{t} \Sigma$ and examine the impact of increasing *t* on the prediction of the random effects.

16 $$\begin{aligned} BLP(u)(y)= & {} t^{-1}\Sigma Z^T(t^{-1}Z\Sigma Z^T + \sigma ^2 I)^{-1}y \end{aligned}$$

17 $$\begin{aligned}= & {} \Sigma Z^T(Z\Sigma Z^T + t \sigma ^2 I)^{-1}y \end{aligned}$$

We want to examine as to whether $e_i^T\Sigma Z^T(Z\Sigma Z^T + t \sigma ^2 I)^{-1}y \ge 0$, i.e., the *i*-th component of the *BLP* is scored above the mean, implies that the derivative (w.r.t *t*) of this expression is negative (ensuring that the BLP shrinks with increasing *t*).

The derivative of $e_i^T\Sigma Z^T(Z\Sigma Z^T + t \sigma ^2 I)^{-1}y$ is given by:

$$\begin{aligned} -\sigma ^2e_i^T\Sigma Z^T(Z\Sigma Z^T + t \sigma ^2 I)^{-2}y. \end{aligned}$$

With these preliminary remarks, we may now formalize the following result:

#### Theorem 5

If $Z\Sigma e_i$ is nonzero and *not* an eigenvector of the matrix $(Z\Sigma Z^T + t \sigma ^2 I)$, then there is a response vector *y* with the following properties:

- The *i*-th component of the *BLP* is scored not below 0, i.e.,
  $$\begin{aligned} e_i^T\Sigma Z^T(Z\Sigma Z^T + t \sigma ^2 I)^{-1}y\ge 0. \end{aligned}$$
- There is a $t'>t$ such that the *i*-th component of the *BLP* is placed further away from zero under $t'$, i.e., we have
  $$\begin{aligned} e_i^T\Sigma Z^T(Z\Sigma Z^T + t' \sigma ^2 I)^{-1}y> e_i^T \Sigma Z^T(Z\Sigma Z^T + t \sigma ^2 I)^{-1}y\ge 0. \end{aligned}$$

#### Proof

According to the preliminary remarks, we may examine conditions under which the inequality $e_i^T\Sigma Z^T(Z\Sigma Z^T + t \sigma ^2 I)^{-2}y\ge 0$ (ensuring a nonpositive derivative at *t*) is a consequence of the inequality $e_i^T\Sigma Z^T(Z\Sigma Z^T + t \sigma ^2 I)^{-1}y \ge 0$. According to Farkas’ Lemma, this holds if and only if there is a nonnegative scalar $\lambda$ such that

$$\begin{aligned} e_i^T\Sigma Z^T(Z\Sigma Z^T + t \sigma ^2 I)^{-2} =\lambda e_i^T\Sigma Z^T(Z\Sigma Z^T + t \sigma ^2 I)^{-1} \end{aligned}$$

or equivalently (transposing both sides and canceling one inverse)

$$\begin{aligned} \lambda Z\Sigma e_i = (Z\Sigma Z^T + t \sigma ^2 I)^{-1} Z \Sigma e_i \end{aligned}$$

The latter equation means that $Z \Sigma e_i$ is an eigenvector corresponding to a nonnegative eigenvalue $\lambda$ of $(Z\Sigma Z^T + t \sigma ^2 I)^{-1}$. As $(Z\Sigma Z^T + t \sigma ^2 I)^{-1}$ and $(Z\Sigma Z^T + t \sigma ^2 I)$ have the same eigenvectors (and reciprocal eigenvalues) the result follows. $\square$

#### Remark

- Due to the symmetry of the involved expressions in $(t, \sigma ^2)$, we may derive the same results for a fixed *t* while increasing $\sigma ^2$. The result depicted in (*a*) and (*b*) may then be interpreted as an increase in the measurement error which amplifies the distance of the *BLP* from its mean.
- By noting that there is a one-to-one correspondence between eigenvectors of $(Z\Sigma Z^T + t \sigma ^2 I)$ and those of $Z\Sigma Z^T$, we may replace the condition underlying Theorem 5 by simply demanding that $Z\Sigma e_i$ is not an eigenvector of the matrix $Z\Sigma Z^T$.

## Simulations Based on Real Data Examples

Though the previous analysis pointed out the existence of responses which imply reverse shrinkage effects, it is by no means clear if the described effect is likely to occur in practical applications of the models. To address this question, we provide for each of the three discussed cases a simulation which is based on parameter estimates obtained within real data settings. The R-code underlying the simulations is provided as supplementary material. Here we focus on describing the most important aspects of the simulation.

### Case 1: Linear Model with Offset

We use the working memory test battery described in Oberauer, Süß, Schulze, Wilhelm, and Wittmann (2000) to illustrate and quantify the described amplification phenomenon. The test battery consists of 25 tasks (items) which serve as the manifest variables of a factor analysis model that is described in table 4 of Oberauer et al. The model contains three orthogonal factors (for an example with correlated factors see case 3 below) labeled as “verbal-numerical,” “spatial,” and “speed” and a (predominantly) nonnegative matrix of factor loadings. The reported communalities allow for the computation of the measurement error variances. These (unequal) measurement error variances are incorporated in the analysis via the weight matrix *W* as mentioned in the generalizing remarks following the discussion of case 1 in the previous section. More specifically, if $c_i$ denotes the communality of the *i*-th item, then $(W)_{ii}:=1/(1-c_i)$ holds. Furthermore, without loss of generality all offsets were set to zero.

In order to gain an impression on the prevalence and magnitude of the described amplification effect we conducted a small simulation using the given factor analysis setup. More specifically, we simulated responses according to the model

$$\begin{aligned} y=X\beta + \epsilon \end{aligned}$$

with *X* denoting the $(25 \times 3)$ matrix of factor loadings, $\epsilon$ denoting the vector of normally distributed measurement error variables with variances $\sigma ^2_{\epsilon _i}=1-c_i$. We repeatedly simulated a draw of a test taker from the population via drawing $\beta \sim N(0,I)$ and then sampling a realization for her responses according to the above equation—via a draw from the specified distribution for the measurement error variables. For the shrinkage penalty we specified a term of the form $t\Sigma ^{-1}$, with $\Sigma =I$ matching the orthogonality of the factor model. For the shrinkage parameter, three levels $t=0$ (MLE, WLS), $t=1$ and $t=2$ were compared. We then quantified the proportion of trials wherein the shrinkage estimate $\left( (X^TWX + tI)^{-1}X^TWy\right)$ contained an entry that is further apart from the prior mean than the corresponding entry of the weighted least-squares estimate ($(X^TWX)^{-1}X^TWy$). Roughly 38% of the simulated responses showed such a behavior, i.e., contained at least one component wherein the amplification effect could be observed. The magnitude of the effect seemed to depend on the exact level of shrinkage. For $t=1$ ($t=2$), the relative magnitude, i.e., $1-\frac{|\hat{\beta }_i|}{|\hat{\beta }^s_i|}$ (with $\hat{\beta }^s$ denoting the shrinkage estimate and $\hat{\beta }$ denoting the weighted least-squares estimator), was 23% (30%), whereas the absolute magnitude, i.e., $|\hat{\beta }^s_i|-|\hat{\beta }_i|$, was .035 (.06), i.e., $3.5\%$ ($6\%$) of one standard deviation ($\sigma _{\beta _j}=1$ for all *j* according to the simulation setup) of the population factor score distribution. In rare cases, the absolute magnitude was as large as 17% (28%) of the standard deviation. Moreover, decreasing the number of manifest variables (i.e., the information provided by the data) increased the magnitude and the prevalence of the amplification effect further.

The conducted simulation study also allowed for the quantification of the shrinkage overshoot effect (Sect. 2.5) by examining sign reversals. Within $6\%$ ($10\%$) of all trials, there was some component *i* with respect to which the estimates differed in sign. The latter means that the shrinkage did not stop “properly” at the prior mean, but extended beyond that mean (see also the introductory example of Bob in Sect. 1).

We close this simulation case by noting that similar results on the prevalence of the amplification effect were observed when comparing only slightly differing shrinkage levels. That is, when we compare the levels *t* and $t+\epsilon$ with $\epsilon$ very small ($\epsilon = 0.001$ was used in the simulation), then again a prevalence estimate of 38% was computed. The supplementary material contains the full R code for reproducibility.

### Case 2: Linear Model with Centered Predictors

As an illustration for the amplification effect in the second case, we use the classical ridge regression setup. The data are reported in Gorman and Toman (1966) and have been part of the classical ridge regression analysis by Hoerl and Kennard (1970a; b). Our focus is, however, not on statistical properties of the estimates, but rather on the outlined shrinkage and amplification effects.

The data include a correlation matrix for a multiple regression with 10 predictors as well as the correlation of each predictor with the dependent variable. For the simulation, we repeatedly generated data according to a multivariate normal distribution (centered at zero and with unit variances) with the given correlations. That is, we (repeatedly) sample $n=100$ observations from a 11-dimensional joint normal distribution with the given correlation matrix. We then centered the dependent variable and compared the MLE ($(X^TX)^{-1}X^T y^c$) with the corresponding shrinkage estimate. The computed estimates (MLE and ridge estimate) were then compared with respect to the amplification property and in addition with respect to any occurrences of sign reversals.

Of course, the results are dependent on the choice of the shrinkage parameter and the size of the data set. The choice $n=100$ and $t=.5$ (within the range of values examined by Hoerl and Kennard) led to roughly 3% sign reversals and 100% amplification cases.^7^ That is, within every simulated data set there was at least one predictor (generally more than one) for which the inferred regression coefficient was larger (in absolute magnitude) under the shrinkage method than under the least-squares method. Moreover, in 3% of all simulated data sets the sign of the inferred regression coefficients differed. For a sketch of the behavior of estimates, when the shrinkage level is continuously increased, we refer the reader to Figure 1 of Hoerl and Kennard (1970b).

### Case 3: Normal Ogive Ordinal Regression Model

As an example of a log-concave likelihood with nonnegative predictors, we use a multidimensional graded response model (MGRM) from item response theory (IRT). In this model, the probability of obtaining score *j* on the *i*-th item for a test taker with latent abilities $\beta$ is given by

18 $$\begin{aligned} P(Y_i=j|\beta )=\Phi (x_i^T\beta -\mu _{i,j})-\Phi (x_i^T\beta -\mu _{i,j+1}), \end{aligned}$$

wherein the parameters $\mu _{i,j}$ constitute ordered thresholds on the latent continuum ($\mu _{i,j}<\mu _{i,j+1}$; see, e.g., ch. 6 in Lee, 2007). It can be deduced that the log-likelihood function, which results from observing the data on *k* items ($y_1, \dots , y_k$) is concave (see example 2.6 in Jordan & Spiess, 2012) and that each term $l_i$ of the log-likelihood satisfies $l''_i<0$.

We use the parameter estimates displayed in Petersen, Groenvold, Aaronson, Fayers, Sprangers and Bjorner (2006) for our simulations. More specifically, Petersen et al. (2006) report the results of fitting a three-dimensional MGRM with the factors “physical functioning,” “fatigue,” and “emotional functioning” to 12 items of a health-related quality of life item pool. The estimated item discrimination vectors $x_i$ are nonnegative and the latent dimensions exhibit strong correlations with each other. Hence, this example provides the opportunity to examine the previously described effects within a different setting that includes correlations in the prior. The following matrix of factor correlations was therefore used:

$$\begin{aligned} R:=\begin{pmatrix} 1 &{} \quad 0.8 &{} \quad 0.45 \\ 0.8 &{} \quad 1 &{} \quad 0.56 \\ 0.45 &{} \quad 0.56 &{} \quad 1 \end{pmatrix} \end{aligned}$$

The simulation proceeded as follows: We defined the loglikelihood using the log of Eq. (18) in place of the quantity $l_i(x_i^T\beta )$ appearing in Eq. (6). The penalty was specified according to the expression $-t\beta ^T \Sigma ^{-1} \beta$ with $\Sigma :=R$ matching the reported correlation matrix of the latent abilities. We then first drew a vector of latent abilities from the population using the reported factor correlation matrix (which equals the factor covariance matrix as all factors are standardized with unit variance). We then simulated a response according to the MRGM and evaluated the effect of inducing shrinkage. The simulation of a response was accomplished via Eq. (18) by computing the response probabilities for each category and then sampling a category using the response probability as a sampling weight. In contrast to the previous cases, to evaluate the shrinkage effect, we did *not* compare the shrinkage estimate with the MLE. Instead, we decided to compare two shrinkage estimators with different levels of shrinkage. This change is due to the fact that the MLE may not exist for a variety of response patterns (this problem becomes less severe with a large number of items though), which may impede the examination of the shrinkage effect. In contrast, the shrinkage estimator exists for any possible response pattern. Hence, by comparing two different levels of shrinkage, the problem of the nonexistence of estimates is alleviated and a comparison is then straightforward. The results of the simulation—using $t=0.5$ and $t=1$ as levels of the shrinkage parameter—are comparable to case 1: In 47% of all simulation trials, we observed an amplification effect (“reverse shrinkage”) on at least one dimension. In addition, approximately 4% of all trials showed a sign reversal, that is, the two shrinkage estimates differed in the sign for some latent dimension.

## Graphical Explanation

The described “amplification effect” may at first sight seem counterintuitive. However, we argue that the counterintuitive aspect is solely invoked by the oftentimes inappropriately applied label of “shrinkage.” In fact, if one subsumes under the label “shrinkage” just the implicit understanding that the *length* of the parameter vector shrinks, then there is nothing odd about the notion that some component might increase in magnitude (though this by no means implies that this behavior does not cause any problems in some applied settings—see the discussion in Sect. 5). However, our experience is that practitioners as well as some researchers expect the shrinkage effect to appear on each component. To provide a graphical explanation of why the latter is not true, it is useful to recast the Bayesian MAP in a Lagrange multiplier framework, which will then furnish a simple geometrical explanation (see also chapter 17 in Draper & Smith, 1998). To this end, suppose that within the linear regression framework we want to minimize the log-likelihood $l(\beta )$ (which basically equals a least-squares problem) under the additional constraint that the length of the regression parameter $\beta$ is bounded by *c*, i.e., under the constraint expressed by $f(\beta )\le 0$ with $f(\beta ):=\beta ^T \beta -c^2$. Then, the estimator under this constraint can be derived by solving the unconstraint optimization problem

$$\begin{aligned} l_p(\beta ):=l(\beta )+\lambda f(\beta )=l(\beta ) +\lambda \beta ^T \beta - \lambda c^2, \end{aligned}$$

wherein $\lambda$ is a nonnegative Lagrange multiplier. The resulting estimator of $\beta$ in this problem equals the Bayesian MAP using the parameter $t=\lambda$ as shrinkage parameter. Hence, shrinkage estimators might be viewed as originating from a constraint optimization problem, wherein the constraint provides a sharp bound on the length of the regression parameter vector. Figure 1 illustrates the amplification effect within this modified, Lagrangian framework: The ellipsoids represent contour lines of the log-likelihood with the MLE (marked in red) in the center of the highest contour. By introducing a sharp constraint $\beta ^T \beta -c^2\le 0$, we effectively search for the intersection of the circle (black) with the highest possible contour. This optimal point is marked in yellow in the figure and it can be seen that the y-coordinate of this point is further away from the prior mean (zero) than the respective MLE-coordinate. In fact, when casted within this framework, no expectations on (componentwise) shrinkage would arise in the first place, because a circle can clearly intersect the highest contour at a point which lies below the maximal contour (consisting of a single point—the MLE). Note that this reasoning has already been given in the context of ridge regression by, for instance, Draper and Smith (1998). However, the potential problem resulting from the amplification effect has—at least to the author’s knowledge—not been noted.

Likewise, using the same rationale, Fig. 2 illustrates the possibility of sign reversals (“shrinkage overshoot”): Whereas the second component (y-coordinate) of the MLE is negative (i.e., Bob’s verbal IQ is estimated below the prior mean), the second component of the corresponding shrinkage estimator is positive (i.e., Bob’s verbal IQ is estimated above the prior mean when we account for the population distribution). Again, from the geometrical viewpoint there is no problem in understanding this effect. Yet, it poses practical challenges (Sect. 5).

## Discussion

In this paper, we demonstrated some less well-known properties of shrinkage estimates—namely “reverse shrinkage” and “shrinkage overshoot”—on both, an analytical level and within simulations based on real data examples. The purpose of the paper was *not*, however, to cast doubt on the usefulness and statistical properties of shrinkage estimators. Rather, the aim was to *a*) depict various misconceptions about the (anticipated) effect of shrinkage and *b*) to highlight that certain by-products of shrinkage estimators, like the amplification effect on some dimensions, might cause problems in (some) applied research settings (these setting are further depicted below).

For instance, though we might just accept the amplification effect as a by-product of a statistically reasonable estimate, the notion that a test taker is placed further away from the population mean when using the estimator might be hard to convey to the practitioner. This holds for high- and low-stakes testing situations, for achievement as well as for personality tests. Further, as the effect depends on the particular observed response pattern, it might very well be the case that the ordering of two test takers is swapped when applying the shrinkage estimator. That is, Bob might score lower on the verbal IQ than John when using the MLE, yet this ordering might be reversed when introducing shrinkage. Hence, the ordering of subjects may differ according to the specific paradigm of factor score estimation. Note that this is true even in the presence of independent priors for the latent dimensions.

Additional problems are caused by the effect of “shrinkage overshoot”: Using the MLE, Bob’s verbal IQ is estimated below average (say 99). Yet, introducing the prior (with mean 100), we end up with a shrinkage estimate above average (101), which might be difficult to justify from an applied viewpoint. In general, however, the simulations showed that the size of “shrinkage overshoot” was relatively small, so that it might cause only problems in specific practical cases, wherein the qualitative movement needs some justification.

It can be useful to cast these phenomena in Bayesian terms (following the Bayesian perspective on ridge regression as, for example, described in Hsiang, 1975). In these terms, the amplification effect may be described as follows: Our prior knowledge specifies unrelated factors and abilities drawn from a population with mean zero. Yet, regardless how strict our knowledge is (determined via setting the common prior precision), there will always be some response vector such that some of the test taker’s abilities is inferred larger under this prior than under a flat (improper) prior. Further, shrinkage overshoot may be described as follows: If we have a test taker with an estimate (assuming a flat prior) above average, then introducing the prior knowledge, i.e., assuming the test taker was drawn from a population with mean zero, lowers his/her estimate toward a value below average. Hence, it can be seen that the described effects are somewhat difficult to convey in terms of the usual Bayesian interpretation. We therefore think that the constrained optimization formulation given in Sect. 4 provides a clearer explanation for the described shrinkage effects.

However, the interpretation of the estimate as a conditional expectation entails that the estimate is optimal in terms of mean square error. This implies that, on average, the accuracy is improved by using the shrinkage estimator (naively assuming the shrinkage term is based on the true prior distribution with no misspecification). The prove of this classical result is given in ch. 7 of Searle, Casella, & McCulloch (2006). Hence, in terms of statistical accuracy, the shrinkage estimator should be the preferred way of estimating the person parameter(s). We note that in the unidimensional case they are valid types of estimates. In fact, they are free from paradoxical scoring (i.e., correct answers are always rewarded) and there is also no implied reversal in the ordering of subjects. However, this changes in the multidimensional setup: Firstly, paradoxical scoring issues may arise, whereby correct answers are penalized on some latent dimensions (Hooker et al., 2009; Hooker, 2010). However, note that this would not provide a valid argument to avoid these types of estimates in all cases because the existence of such paradoxical scoring patterns is dependent on the particular type of prior distribution. That is, for some type of priors (see Hooker, 2010) paradoxical scorings do not occur regardless of the observed scoring pattern. If the true prior coincides with one of these types of priors, then, by the same argument as stated above, optimal and valid estimates are obtained by using the shrinkage estimator. Secondly though, the results presented in this paper clearly show that there is yet another important property which should be taken into account before adopting these types of estimates into practice—namely the reverse shrinkage effect. As already explained, this can cause a further challenge in test score interpretation and justification.

It should, however, be emphasized that the above statement on the statistical efficiency must be treated with great care. That is, only in the case of a correct prior specification (i.e., the prior coincides with the true distribution of the latent abilities in the population), the results on optimality hold. Hence, two necessary conditions for the proper application of the EAP estimates immediately arise. Firstly, the proposed model should fit the data of the test construction (calibration) well. If this is the case, then there is also some evidence that the presumed normality distribution of the latent abilities holds, because otherwise the test calibration data would already highlight some misfit. This owes to the fact that for the fit of an IRT model not only the correct specification of the item response functions is necessary, but—with the rare expectation of Rasch models—also the distribution of the latent variables has to be specified correctly. Therefore, given a decent model fit during test construction, a second prerequisite for the application of the EAP demands that the population with respect to which the test is applied does only show minor differences from the population with respect to which the test was constructed.

However, even in the benevolent case of a correct specification, the EAP estimates usually introduce (additional) statistical bias, which only vanishes in very long tests. Although there are methods to reduce the bias (Bock & Mislevy, 1982), the general issue remains. This sort of bias is different from the bias that results from paradoxical scoring, but it may likewise entail problems with the fairness of the scoring—due to the non-uniformity of the bias across the ability levels. There are therefore multiple objections for the adoption of these estimates in the scoring of individual test takers which underscore their rare usage for the purpose of psychological and educational diagnostics. This limits the applicability of the results of the paper to (*i*) research cases (examples are given below); to (*ii*) practical applications of multidimensional adaptive testing, where there is a prime emphasis on obtaining statistical optimal estimates and (*iii*) subscore reporting as addressed by Haberman and Sinharay (2010). The latter paper also outlines some interesting applications of the (EAP-based) subscore reporting:

“Failing candidates want to know their strengths and weaknesses in different content areas to plan for future remedial work. States and academic institutions such as colleges and universities often want a profile of performance for their graduates to better evaluate their training and focus on areas that need instructional improvement” (p. 209 in Haberman & Sinharay, 2010)

In general the outlined problems only arise when shrinkage estimators are applied in practice and when the test is multidimensional. Many tests in practice are constructed with the specific aim to achieve unidimensionality or simple structure. These tests are then scored in terms of monotone scores (in most cases: simple sum scores on each dimension), even if they do not adhere to unidimensionality in a strict sense, i.e., there may exist some nonzero cross-loadings in the matrix of factor loadings. They are therefore not prone to any of the described phenomena. However, there are at least two unsatisfactory issues with this common practice. Firstly, we construct scales according to psychometric theory to which the efficient estimation of a test taker’s latent ability is of prime concern (in fact, this is the main point underlying the psychometric theory of adaptive testing). Yet, the last stage in this process—namely the estimation of the person parameters—seems to differ from the strict adherence to statistical methodology in that oftentimes practitioners refrain from applying a textbook statistical estimate (MLE/Bayes) based on a complete model. By the latter, we specifically mean that any realistic test battery, will show some cross-loadings (even if constructed with a specific focus on unidimensionality) and—perhaps even more importantly—will also consist of correlated latent dimensions. Hence, common statistical reasoning would suggest that (*a*) incorporating the cross-loadings in the model^8^ and (*b*) taking into account the statistical association between the latent dimensions could only improve the accuracy of the estimates (this can be made rigorous via examining the area of confidence regions and via examining the MSE). Thus, from the perspective of statistical efficiency, it may very well be deduced that the common practice should move toward the incorporation of the full factor loading structure and of factor correlations. This ultimately means that the test would be scored as a multidimensional test with prior information (on the factor correlations) to which all the results on the shrinkage effects apply. In fact, arguments for the incorporation of shrinkage estimator in the tailored testing framework based on considerations of optimality were already given by Owen (1969).

We also note that, as psychometric theory advances, a trend toward more complex models is occurring. For example, the topic of MIRT is a relatively new one (Reckase, 1997), yet significant research interest has been devoted to the advancement of MIRT models. Additionally, topics like multidimensional adaptive testing (Segall, 1996) clearly show that these models aim at improving the classification and diagnoses of test takers. In some cases, direct (multiple hurdle) rules based on multidimensional scores are considered (Segall, 2000). In other cases, the scores derived from multidimensional models are used to derive performance profiles. For example, Luecht (1996) discusses the use of multidimensional scores in the context of a medical certification test. For the university and educational context, Haberman and Sinharay (2010) point out that multidimensional (EAP based) scores may be used to evaluate the training of students and to identify areas which need improvement. A trend toward the incorporation of the more realistic multidimensional models can also be seen in refinements of previously unidimensionally scored scales. A good example is the Law School Admission Test (LSAT). As already noted by Bock and Lieberman (1970), for some subtests of the LSAT7, the hypothesis of unidimensionality is questionable. Wainer (1994) provided the testlet model as an improvement over the simple unidimensional model. As the latter boils down to a multidimensional model with correlated latent dimensions, we have an example of a test within a high-stakes testing framework which was not intentionally designed multidimensional, but upon closer examination turns out to be multidimensional. A similar example of a high-stakes testing framework is provided by the Armed Services Vocational Aptitude Battery, wherein the correlations among the latent dimensions are given in Table 2 of Segall (2001). Further, the scoring algorithm described by Segall (2001) employs unidimensional shrinkage—and a rationale for the incorporation of the significant correlations between the latent dimensions is provided in Segall (1996).

Further, many testing setups naturally require multidimensionality. The simultaneous modeling of accuracy and speed in tests with a speed component either requires a model wherein the latent parameters may not be seperated (e.g., a drift-diffusion model; see van der Maas et al., 2011), or a hierarchical response time model (van der Linden, 2007) which requires specification of a joint prior for the latent variables with nonzero correlations. Likewise, the measurement of change in a longitudinal setup requires the specification of a joint (prior) distribution of the ability and the change parameter(s). As an alternative, the inference of the latent abilities on the first time point might be treated as prior knowledge for the inference on the subsequent time point.

In conclusion, this sketches a multitude of test settings wherein the behavior of multidimensional shrinkage estimators is of interest. The counterintuitive results on shrinkage in the multivariate case as outlined in this paper provide a novel perspective with respect to which these more complex models should be evaluated in future research—especially when these models are ultimately used for diagnostic and classification purposes. Additionally, extensions of the outlined results toward other types of shrinkage penalties (e.g., $L_1$ loss) can be of interest and the graphical interplay of the penalty term with the likelihood contours, as sketched in Sect. 4, can furnish a useful approach in the analysis of these extensions.

Finally, there is another subtle (but important) by-product of the introduction of the penalty/prior term, which has not been the focus of this paper, but which has been previously discussed in the context of paradoxical scoring effects in multidimensional item response theory models (see Hooker et al., 2009; Hooker, 2010): The scoring direction of each individual item may change. That is, if, for example, higher scores on item 1 increase the numerical IQ estimate when using MLE scoring, it might very well be the case that higher scores on the very same item lead to decreases in the numerical IQ estimate when using the shrinkage estimator (of course, the reverse is also possible, hence this should *not* be read as an argument in favor of using MLE instead of MAP estimates). Thus, whether a test taker is penalized or rewarded for a better performance on an item depends on the applied type of ability estimation (MLE or Bayes). For a more detailed discussion of this topic, we refer the reader to the appendix, wherein we describe the analysis of this effect in a two-dimensional setting.

Therefore, we want to point out that statistically reasonable estimates can (still) pose problems and challenges when considered under additional criteria (e.g., test fairness in the domain of educational testing) which may be highly relevant for the application at hand. Moreover, we view the described effects as providing yet another example, wherein the intuition developed from one-dimensional models leads us astray in higher dimensional models. That is, we may very well have a reasonable explanation for a statistical effect in the one-dimensional setting (e.g., the explanation of the shrinkage effect in the IQ testing example with a single dimension), yet the very same method of explanation seems questionable and not applicable when used within two- or higher dimensional settings.

Overall we do not want to overemphasize the potential problems of the outlined effects. There may very well be a multitude of multidimensional tests wherein the described phenomena do either not pose any threats (because of the way the scores are used) or are diminished in their magnitude due to a long test length. We have sketched three areas (see (*i*), (*ii*), (*iii*) above), wherein we think it is very helpful to be aware of the problems posed by applying shrinkage estimators. The existence of these effects in conjunction with the already established results on paradoxical scoring behavior point to the necessity to formulate explicit requirements for the behavior of person parameter estimates in terms of *non-statistical* properties. That is, for reasons of fairness one may require monotonicity of the scoring in each item. Likewise, one may safeguard against the effects discussed in this paper by requiring “similar” scoring behavior across the multiple dimensions when introducing prior knowledge. Taken together with other important notions (such as, for example, the sensitivity to extreme responses) these requirements may serve as an additional checklist which adds additional aspects to the (usually predominantly statistically driven) choice of estimates for the inference of the latent abilities.

### Supplementary Information

Below is the link to the electronic supplementary material.

Supplementary file 1 (csv 0 KB)

Supplementary file 2 (csv 0 KB)

Supplementary file 3 (txt 0 KB)

Supplementary file 4 (txt 0 KB)

Supplementary file 5 (txt 2 KB)

Supplementary file 6 (txt 1 KB)

Supplementary file 7 (txt 2 KB)

## A Farkas’ Lemma

The statement of the following Lemma can be found in many textbooks on convex analysis. As it is frequently used in the proofs, we restate it here. To introduce the central notation, let $(a_i)_{i=0, \dots m}$ denote fixed vectors in ${\mathbb {R}}^n$. By definition, we say that an inequality $a_0^T x \le 0$ is a consequence of the system of inequalities $(a_i^T x\le 0)_{i=1, \dots m}$ if and only if every *x* satisfying $a_i^T x \le 0$ for $i=1, \dots m$ also satisfies $a_0^T x \le 0$. With this terminology in mind, Farkas’ Lemma can be stated as follows (adapted from Corollary 22.3.1 in Rockafellar, 1970):

### Lemma 1

(Farkas’ Lemma) An inequality $a_0^T x \le 0$ is a consequence of the system

$$\begin{aligned} a_i^T x\le 0, \qquad i=1, \dots m \end{aligned}$$

if and only if there exist non-negative real numbers $\lambda _1, \dots \lambda _m$ such that

$$\begin{aligned} \sum _{i=1}^m \lambda _i a_i = a_0. \end{aligned}$$

Note that the equation appearing in Farkas’ Lemma is equivalent to stating that the vector $a_0$ is expressible as a non-negative linear combination of the set of vectors $(a_i)_{i=1, \dots m}$.

## B Dependency of the Scoring Direction on the Shrinkage Parameter—the Two-Dimensional Case

In the discussion, we raised a question concerning the influence of the penalty parameter *t* on the scoring direction of an item. In this appendix, we provide an analytic view on the topic. More specifically, given a two-dimensional test of the linear model type (i.e., modeling assumptions in accordance with case 1) with positive predictors, we examine as to whether there exist two different shrinkage levels, $t' \ne t$, such that an item is scored in different directions under these shrinkage levels. That is, increasing the item score increases the latent ability estimate when the shrinkage level $t'$ is used, the same increase lowers the corresponding estimate when the shrinkage level *t* is used.

To this end, recall the expression for the shrinkage estimator:

$$\begin{aligned} \hat{\beta }(t)=(X^TX + tI)^{-1}X^Ty. \end{aligned}$$

Without loss of generality, we will restrict the following discussion to the first component of $\hat{\beta }(t)$. We will further restrict ourselves to the discussion of a compensatory test pattern, that is, we will assume an entry-wise positive design matrix. In the psychometric framework, the latter corresponds to the notion that each latent ability contributes positively to the solving of each item.

The scoring direction of item *j* with respect to the first dimension is determined by the sign of

$$\begin{aligned} e_1^T (X^TX + tI)^{-1}X^Te_j = e_1^T (X^TX + tI)^{-1}x_j, \end{aligned}$$

wherein $x_j$ denotes the *j*-th row of the design matrix (considered as a column vector).

For the two-dimensional setting we further have

$$\begin{aligned} X^TX=\begin{pmatrix} a &{} \quad b \\ b&{} \quad d \end{pmatrix}, \quad X^TX + tI =\begin{pmatrix} a+t &{} \quad b \\ b&{} \quad d+t \end{pmatrix}, \end{aligned}$$

from which an expression for the inverse $(X^TX + tI)^{-1}$ may directly be deduced as

$$\begin{aligned} (X^TX + tI)^{-1}=f(t)\begin{pmatrix} d+t &{} \quad -b \\ -b&{} \quad a+t \end{pmatrix}, \end{aligned}$$

with *f*(*t*) denoting a strictly positive function—only depending on the determinant of the matrix $X^TX + tI$. As *f*(*t*) is strictly positive, we may drop it for the sake of analyzing the sign of the expression $e_1^T (X^TX + tI)^{-1}x_j$. Hence, the sign is determined by the formula

$$\begin{aligned} e_1^T \begin{pmatrix} d+t &{}\quad -b \\ -b&{} \quad a+t \end{pmatrix} x_j =\begin{pmatrix} d+t&\quad -b \end{pmatrix} \begin{pmatrix} x_{j1} \\ x_{j2} \end{pmatrix}=dx_{j1}+tx_{j1}-bx_{j2}. \end{aligned}$$

Accordingly, as $x_{j1}> 0$, we have

$$\begin{aligned} e_1^T (X^TX + t_0I)^{-1}x_j = 0 \end{aligned}$$

for the choice

19 $$\begin{aligned} t_0:=\frac{bx_{j2}-dx_{j1}}{x_{j1}}. \end{aligned}$$

Note that choosing *t* according to Eq. (19) leads to an estimate which is insensitive to changes of the score on the *j*-th item. Further, choices $t'>t_0>t$ lead to different scoring directions. That is, assuming $x_{j1}>0$, higher scores on the *j*-th item are rewarded when the level $t'$ is used, whereas higher scores are penalized when the level *t* is used. However, currently this just refers to a hypothetical scenario as the value $t_0$ computed in (19) may be negative and may thus not correspond to a valid choice of a (necessarily positive) penalty parameter. We therefore need to examine the sign of $t_0$ more closely.

To this end, note first that for a valid choice of $t_0$ we must have $bx_{j2}-dx_{j1}>0$. We will show that there is at least one item *j* fulfilling this condition. We argue by contradiction. Therefore, assume that $bx_{j2}-dx_{j1}\le 0$ holds for all choices of *j*. Multiplying both sides by $x_{j2}$ and summing over *j* then leads to:

$$\begin{aligned} b\sum _{j}x_{j2}^2 \le d \sum _{j}x_{j1}x_{j2}. \end{aligned}$$

According to the definitions (recall that *b* and *d* are defined via inner products of columns of *X*), we further have $b=\sum _{j}x_{j1}x_{j2}$ and $d=\sum _{j}x_{j2}^2$. Hence, it follows that

$$\begin{aligned} bd \le d b \end{aligned}$$

which can only be true if all inequalities aren’t strict, i.e., if $bx_{j2}-dx_{j1}= 0$ for all *j*. The latter implies $bx_{j2} = dx_{j1}$ for all *j*. Assuming $b\ne 0$ (which is implied by the assumption of a positive design matrix), we therefore have derived a linear dependence between the columns of *X*. This result contradicts the assumption of a design matrix of full column rank. Therefore, it follows that there exists at least one *j* such that $bx_{j2}-dx_{j1}>0$ holds. Moreover, for this *j* we can obtain a valid $t_0$ via (19) with all the implications that were outlined in the previous paragraph.

### Theorem 6

Let *X* denote a $(k \times 2)$ matrix with rank 2 and strictly positive entries. Let $\delta _{1,j}(t):=e_1^T(X^TX+tI)^{-1}X^Te_j$ denote the scoring direction of scoring the *j*-th item with respect to the first dimension. Then there is an item $j^*$ and a positive penalty parameter $t_0$ such that for $t'>t_0>t$

$$\begin{aligned} \delta _{1,j^*}(t')>0, \, \, \delta _{1,j^*}(t_0)=0, \, \, \delta _{1,j^*}(t)<0 \end{aligned}$$

holds.

Stated in Bayesian terms: There is some level of prior knowledge such that the score on the item is irrelevant with respect to the estimate of the first component. For stronger priors, the item is scored positively, whereas for weaker priors (controlled by the magnitude of *t*) the item is scored negatively.

### Remark

For the related topic of the discussion on different prior specifications on (paradoxical) scoring directions of items, i.e., for the examination of which choices of prior correlations $\Sigma$ enable paradoxical scoring, we refer the reader to Hooker (2010). Note that the analysis of Hooker is concerned with the deduction of paradoxical scoring given a fixed $\Sigma$ (not necessarily diagonal), whereby paradoxical scoring means that increases in the item score lead to decreased estimates for some latent dimension. In contrast, the previous analysis was concerned with changes in the scoring direction when the precision of prior information is changed.

## C Relation to Results on Paradoxical Scoring in Multidimensional Latent Variable Models

Readers familiar with the paradoxical scoring effect in multidimensional IRT models (Hooker, Finkelman & Schwartzman, 2009) might wonder as to whether there is a close connection between the outlined results on “paradoxical shrinkage” and the paradoxical scoring effect. In this appendix, we clarify similarities and differences of these two phenomenons.

We thereby need to distinguish between two levels: A semantic level, referring to the meaning of these effects, and a technical level which “only” deals with similarities in the mathematical approach to deduce these effects.

### Semantic Level

It will be helpful to recall first the main parameters of the underlying mathematical model. In a very broad way, the responses of a test taker to the items of a multidimensional test determine a loglikelihood function $l(\beta |y)$. The latter is composed of individual item contributions $l_i$, that is, (due to the local independence assumption) we have the decomposition $l(\beta |y)=\sum _{i=1}^k l_i(\beta |y)$. Depending on the type of inference (i.e., Bayesian vs. Likelihood), we also incorporate a log-prior with the strength of prior information controlled by a (scalar) shrinkage parameter *t*. The resulting function, which forms the basis for statistical inference, is denoted $f(\beta , y,t)$ with the corresponding maximizer denoted by $\hat{\beta }(y,t)$. The purely loglikelihood-based inference is included in this definition via the relation $f(\beta , y,0)=l(\beta |y)$ with MLE given by $\hat{\beta }(y,0)$.

The paradoxical scoring effect describes the phenomenon that increasing the score on an item decreases the estimate of at least one latent ability—despite the assumption that each latent dimension contributes positively to the solving of each item. Within the factor analysis model used in Sect. 2, the latter may be formalized by a loading matrix containing solely nonnegative entries (Jordan and Spiess, 2012). We emphasize that in the study of paradoxical scoring the shrinkage parameter, if present at all, is fixed.^9^ The interest focuses on studying changes of the response $y_i$ on the estimates for the latent abilities. Formally, for ordered response vectors $y'>y$ (i.e., $y'_i\ge y_i$ $\forall i$ and $y'_j>y_j$ for some *j*) we compare $\hat{\beta }(y',t)-\hat{\beta }(y,t)$ and conclude that this difference contains some negative component. The latter implies that the test taker with a “worse” performance (*y*) obtains a higher ability estimate on some dimension. Of course this raises questions on test fairness which are further described in Hooker et al. (2009) and which were also discussed in later generalizations of the effect (Jordan & Spiess, 2012; van der Linden, 2012; van Rijn & Rijmen, 2015).

To summarize some key aspects of the paradoxical scoring effect:

- The comparison focuses on response changes.
- With respect to the estimates, the magnitude of $\hat{\beta }$ is of no concern in the derivations. The qualitative comparison as to whether we have $\hat{\beta }_i(y',t)-\hat{\beta }_i(y,t)<0$ for some component *i* matters.
- The shrinkage parameter is absent in most derivations of the effect. If the shrinkage parameter is present, then it is fixed.
- In order for the effect to be labelled as paradoxical, some notion of nonnegative item discrimination is paramount. In most modeling classes, the latter boils down to requiring a model with a nonnegative loading matrix (or the IRT counterpart of a matrix of nonnegative item discrimination vectors).
- Typically, the paradoxical effect holds irrespective of the specific response pattern. That is, solely based on the loading matrix *X*, items can be identified which are always scored paradoxically (see Jordan & Spiess, 2018).
- The effect describes a potential practical fairness issue, which arises when comparing the performance of two test takers.

On the other hand, the examinations of the paradoxical shrinkage effects described in this paper do not study changes in *y*, but rather are focused on studying changes in the shrinkage parameter on the magnitude of latent ability estimates. Formally, we examine $\hat{\beta }(y,t')$ and $\hat{\beta }(y,t)$ for $t'>t$. More specifically, we primarily compare the magnitude of each component of $\hat{\beta }(y,\cdot )$ under both shrinkage levels and derive an amplification effect, if some component exhibits a lower magnitude under the lower shrinkage level. Note that there is no intend on comparing two test takers with different performances (the central aspect underlying the paradoxical scoring effect) and also no reason to impose restrictions on the modeling class via postulating a nonnegative loading matrix (as the generalization in Theorem 2 highlights).

To summarize some key aspects of the paradoxical shrinkage effect(s) (i.e., primarily the amplification effect, but most comments are also valid for the “shrinkage overshoot” effect)

- The comparison focuses on changes of the penalty term *t*—responses *y* are fixed.
- With respect to the estimates, the comparison of the magnitudes of $\hat{\beta }_i$ is of prime concern in the derivations (except for the “shrinkage overshoot” phenomenon).
- In order for the effect to be labeled as paradoxical, we do not need the modeling assumption of compensation in the latent abilities, i.e., it is not required that each item discriminates positively on each dimension.
- The paradoxical shrinkage effect typically can only be deduced for some specific responses *y*.
- The effect describes a potential practical issue when comparing the dependency of a test taker’s latent ability estimate on the prior knowledge (i.e., stronger prior knowledge is reflected by a higher choice of the shrinkage parameter).

### Technical Level

The derivations underlying the shrinkage effects, as described in Sect. 2, are based on Farkas’ Lemma (or more generally: on results referring to separations of convex sets). On the other hand, the derivations of paradoxical scoring results, make use of the condition of negative mixed second derivatives of the loglikelihood function (or variants thereof—like depicted in Jordan & Spiess, 2012) and of the presence of an item measuring a single dimension (Hooker et al., 2009). As such, there is no direct mathematical connections between these approaches.

However, one may attempt to artificially embed our shrinkage scenario in the paradoxical scoring scenario as follows (we will highlight below, that at some point, this approach fails): In a purely formal way, we may view the penalty term $-t \sum _i \beta ^2_i$ as the loglikelihood contribution of an additional item. That is, we compare a test taker with corresponding loglikelihood $l(\beta |y)-t \sum _i \beta ^2_i$ with another test taker with corresponding loglikelihood $l(\beta |y)-t' \sum _i \beta ^2_i$. Now, if we impose the restriction of a positive loading matrix, then it can be shown that each loglikelihood function satisfies the fundamental condition of negative mixed second derivatives. However, in order that the proof of the results on paradoxical scoring carries over^10^ to the present context, it would also be necessary that the difference of the two log-likelihood functions depends on a single latent dimension only—which is not the case as we have: $l(\beta |y)-t' \sum _i \beta ^2_i - (l(\beta |y)-t \sum _i \beta ^2_i) = (t-t')\sum _i \beta ^2_i$. The latter is a function of all components of $\beta$. Therefore, to the best of our knowledge, the potential of applying known results on paradoxical scoring to the study of shrinkage effects is confined to a very small special case—namely the special case in which this function reduces to a function of a single component (see Sect. 2.4 on flat priors). Finally, we also note that the mere dependency of the shrinkage effect on the response pattern also points toward this limited transferability. For suppose we could apply the reasoning underlying the derivation of paradoxical scoring. Then, we would have to conclude that the effect holds irrespective of the response pattern *y* which apparently is not true.

## Footnotes

1 This is not to be confused with the estimation of the factor loadings and error variances, which generally rests on treating the factors as random variables rather than as parameters.

2 In general, an inequality of type $a^T_0y\le 0$ is said to be a consequence of a system $(a_i^Ty\le 0)_{i=1, \dots I}$ of inequalities if and only if every vector *y* satisfying the system of inequalities also satisfies the inequality $a^T_0y\le 0$.

3 In the psychometric literature, this type of models, when supplemented with the assumption of nonnegative $x_i$ vectors, is also known as the class of linearly compensatory models—see, e.g., Hooker, Finkelman and Schwartzman (2009).

4 The derivation for the case $p>2$ follows along the same lines.

5 In fact, it is immaterial as to whether we use an improper prior or a proper uniform prior, provided the support of the latter contains a sufficiently large interval centered around zero.

6 In practice, there is oftentimes a two-stage approach: At first, estimates of the unknown components of $\Sigma _u$ and $\sigma ^2$ are derived according to ML-equations. In the following, these estimates are then plugged into the equation determining the *BLP*.

7 We note that a breakdown according to the variables showed that there was a large variation, in that some variables were rarely affected by the effect, whereas on other variables the effect occurred very frequently.

8 Or stated differently: Scoring a test with cross-loadings via ignoring the loadings can in statistical terms be viewed as applying an estimate corresponding to a misspecified model—namely a model wherein the nonzero loadings are set to zero.
